# Inhibition of Specificity Protein 1 Is Involved in Phloretin-Induced Suppression of Prostate Cancer

**DOI:** 10.1155/2020/1358674

**Published:** 2020-08-10

**Authors:** Dan Kang, Wenren Zuo, Qingxin Wu, Qingyi Zhu, Ping Liu

**Affiliations:** ^1^College of Life Sciences, Nanjing Normal University, Nanjing, Jiangsu 210023, China; ^2^Central Laboratory, Jiangsu Province Hospital of Chinese Medicine, Affiliated Hospital of Nanjing University of Chinese Medicine, Nanjing, Jiangsu 210029, China

## Abstract

Phloretin is a flavonoid existed in various plants and has been reported to possess anticarcinogenic activity. However, the anticancer mechanism of phloretin in prostate cancer (PCa) remains unclear. Here, our *in vitro* and *in vivo* experimental data demonstrate that phloretin inhibits the phosphorylation and the activation of EGFR and then inhibits its downstream PI3K/AKT and MEK/ERK1/2 pathways in PCa cells. Inhibition of these two pathways further decreases expression of Sp1 by inhibiting *Sp1* gene transcription, induces degradation of Sp1 protein by inhibiting GSK3*β* phosphorylation, suppresses nucleolin-enhanced translation of Sp1 mRNA by inhibiting nucleolin phosphorylation, and directly inactivates transcription activity of Sp1. Inhibition of Sp1 subsequently decreases the expression of Sp3/4, VEGF, and Survivin and then upregulates apoptosis-related proteins and downregulates cell cycle-related proteins in PCa cells. Finally, phloretin treatment in PCa cells induces cell growth inhibition and apoptosis, suggesting that phloretin may be an effective therapy compound in the treatment of prostate cancer.

## 1. Introduction

Prostate cancer is a commonly diagnosed cancer and the fifth leading cause of cancer deaths in men in the world [1]. Chemoprevention is a promising approach in prostate cancer research, in which natural or synthetic compounds are often used to prevent this malignant disease [2]. Phloretin, a natural flavonoid found mostly in plants [3, 4], has been reported to possess anticancer activity by inducing apoptosis in human glioblastoma cells, Hep G2 cells, and lung carcinoma cells [5–7], while its anticancer molecular mechanism on prostate cancer is still not well known.

Specificity protein (Sp) transcription factors (Sp1/Sp3/Sp4) are often overexpressed in colon cancer, pancreatic cancer, bladder cancer, breast cancer, prostate cancer, and many other cancers [8–12]. The importance of Sp transcription factors (Sps) as drug targets is due to not only their overexpression in multiple cancers but also their relatively low expression in noncancer human tissues [13–15]. Sp-targeted genes are all important in many cellular physiological processes including cell proliferation (such as Sps, AR, and Cyclin D1), cell survival (such as XIAP and Survivin), and angiogenesis (such as VEGF) [16–19].

The PI3K/AKT and MEK/ERK1/2 signal pathways play the crucial roles in cancer cell survival, growth, migration, and invasion [20, 21]. Activation of the PI3K/AKT pathway upregulates the levels of AKT-mediated Sp1 phosphorylation and the activity of Sp1 [22, 23]. Also, activation of AKT inhibits GSK3*β* by increasing the levels of AKT-mediated GSK3*β* phosphorylation. GSK3*β*-mediated Sp1 phosphorylation at Ser728/Ser732 is critical in promoting Sp1 protein ubiquitination and degradation [24]. In addition, ERK-phosphorylated Sp1 at T739/T453 enhances Sp1 binding to DNA and then increases Sp1 transcriptional activity [25–27].

Nucleolin, a multifunctional protein localized not only in the nucleus but also in the cytoplasm and cell membrane, plays an important role in many cellular processes, such as chromatin remodeling, translation of mRNA, transcription of ribosomal RNA (rRNA), rRNA maturation, and ribosome assembly [28]. Usually, nucleolin binds to the G-rich sequence in 3′- or 5′-UTR of target mRNAs and then enhances the translation of target mRNAs [29]. Phosphorylation of nucleolin mediated by VEGF (at Thr76 and Thr84) and EGFR (at Thr641/Thr707) plays a crucial role in binding to and enhancing the translation of target mRNAs [30, 31].

In this study, phloretin treatment in PCa cells decreases the autophosphorylation level of EGFR and its activity and then inhibits its downstream PI3K/AKT and MEK/ERK1/2 pathways. Inhibition of these two pathways subsequently suppresses Sp1 activity by decreasing the expression of *Sp1* gene, enhancing the degradation of Sp1 protein, decreasing the translation of Sp1 mRNA, and reducing the DNA-binding of Sp1, and then results in the downexpression of Sp1-targed *Sp3/4*, *VEGF*, and *Survivin* genes. Finally, the levels of Bax, cleaved Caspase-3/-8/-9, and cleaved PARP-1 are upregulated, while the levels of XIAP, Cyclin B1, and Bcl-2 are downregulated, and cell growth inhibition and apoptosis are induced by the treatment of phloretin in PCa cells *in vitro* and *in vivo*.

## 2. Materials and Methods

### 2.1. Cell Lines, Chemicals, Antibody, and Plasmids

Human prostate cancer cell lines (LNCaP, CWR22Rv1, PC-3, and DU145) and normal prostate epithelial cell line (WPMY-1) were purchased from American Type Culture Collection (ATCC) (Manassas, VA, USA). Cell lines were cultured in RPMI 1640 (Wisent, Nanjing, China) containing 10% FBS (BRL-GIBCO Co. Ltd., CA, USA), penicillin (100 U/ml), and streptomycin (100 mg/ml) (Sigma-Aldrich, St. Louis, MO, USA). Cells were maintained at 37°C in the presence of 5% CO_2_.

Phloretin (>98%), 5-fluorouracil (5-FU), and CHX (cycloheximide) were purchased from Sigma-Aldrich (St. Louis, MO, USA). CCK-8, MTT (3-(4,5-dimethylthiazol-2-yl)-2,5-diphenyltetrazolium bromide), and MG132 (proteasome inhibitor) were purchased from Beyotime Biotechnology (Shanghai, China). Phloretin and 5-FU were dissolved in DMSO and stocked at -80°C, and the stock solutions were diluted in 1× PBS for *in vivo* experiments. TRIzol was purchased from Invitrogen (Carlsbad, CA, USA) and the 5× PrimeScript TM RT-PCR system was from Vazyme Biotech (Beijing, China).

Antibodies of Sp1, VEGF, Survivin, androgen receptor (AR), XIAP, PARP-1, Caspase 3, Cyclin D1, Cyclin B1, AKT1/2/3, EGFR, p-EGFR(Tyr1173), and *β*-actin were purchased from Santa Cruz Biotechnology (Santa Cruz, CA, USA). Antibodies of Sp3/4, tubulin, H3, p-Sp1(Thr453), p-AKT(Ser473), p-ERK1/2(T202/Y204), ERK1/2, and p-AKT(Ser308) werepurchased from Bioworld Technology Inc. (Minneapolis, MN, USA). Antibody of p-PI3K was purchased from Cell Signaling Technology (Boston, MA, USA). Antibodies of Caspase8/p18, Caspase9/p35/p10, Bcl-2, BAX, RAF1, GSK3*β*, PI3K p85(alpha), nucleolin, p-Sp1(Thr739), and Ki-67 were purchased from Proteintech Group Inc. (Wuhan, China). Antibodies of p-GSK3(Ser9), p-C-RAF(Ser338), p-MEK1/2(Ser217/Ser221), and MEK1/2 were purchased from Affinity Biosciences Inc. (Cambridge, UK). Antibodies of p-Nucleolin(Thr76) and p-Nucleolin(Thr84) were purchased from Abcam Company (Cambridge, UK). Goat anti-rabbit IgG-horseradish peroxidase (HRP) and goat anti-mouse IgG-horseradish peroxidase were purchased from Santa Cruz Biotechnology (Santa Cruz, CA, USA).

Nucleolin expression plasmid (pcDNA3.1(+)-nucleolin) and all luciferase reporter plasmids of Sp1-targeted genes, including pSp1(-751/-20)-luc with *Sp1* promoter inserts (-751 bp to -20 bp, including four Sp1-binding sites, detailed in [32]), pSp3(-417/-38)-luc with *Sp3* promoter inserts (-417 bp to -38 bp, including two binding sites in -185 bp/-165 bp, detailed in [33]), pVEGF(-2018/+50)-luc with *VEGF* promoter inserts (-2018 bp to +50 bp, including two binding sites in -89 bp/+50 bp, detailed in [22, 23, 27]), and pSurvivin(-269/-39)-luc with *Survivin* promoter inserts (-269 bp to -39 bp, including two binding sites in -153 bp/-148 bp and -140 bp/-127 bp, respectively, detailed in [34]) were constructed by our lab.

### 2.2. MTT Assay and CCK-8 Assay for Cell Viability and Proliferation

It mainly referred our previous report [35]. In detail, cells were seeded in a 96-well plate at a density of 1 × 10^4^ cells/well overnight and treated with different concentrations of phloretin (0, 20, 50, and 100 *μ*M) for 24 h, and then culture medium was removed and fresh medium (100 *μ*l) was added with 10 *μ*l of MTT (5 mg/ml) or 5 *μ*l of CCK-8 solution. The plate was incubated at 37°C for 4 h in the dark. For CCK-8 assay, the absorbance of the incubations was measured using a microplate reader (Thermo Scientific, Fremont, CA, USA) at 450 nm. For MTT assay, the medium was removed again, and 100 *μ*l of DMSO was added to each well and the absorbance at 570 nm was measured by a microplate reader (Thermo Scientific, Fremont, CA, USA).

All the measured OD values were converted into cell viability and compared with the value of the control well.

### 2.3. Cell Apoptosis Analysis by Flow Cytometry and DAPI Staining Assay

Cells cultured in 6-well plates were treated with phloretin (0, 20, 50, and 100 *μ*M) for 24 h and then harvested for flow cytometry analysis (cell apoptosis assay) by using an Annexin V-FITC Apoptosis Detection Kit (Keygentec, Nanjing, China) according to the instruction of manufacturer. Flow cytometry analysis for cell apoptosis was performed using the EPICS Elite ESP high-performance cell sorter (Coulter Electronics, Ltd., England, UK) and analyzed by ModFit LT (version 2.0; Verity Software), and a minimum of 30,000 events were collected for each sample.

For DAPI staining assay, cells cultured in 12-well plates were incubated with phloretin (0, 20, 50, and 100 *μ*M) for 24 h. Cells were briefly washed with 1× PBS and fixed in 4% formaldehyde for 15 min, and then washed three times with 1× PBS and permeabilized in 0.2% Triton X-100 for 15 min. Finally, cells were stained with DAPI (1 *μ*g/ml) at 37°C for 30 min in the dark and then observed and photographed by fluorescence microscopy (Nikon, IX-71, Japan).

### 2.4. Cell Cycle Analysis by Flow Cytometry

Cells seeded in 6-well plates were treated with phloretin (0, 20, 50, and 100 *μ*M) for 24 h. Cells were harvested for flow cytometry analysis (cell cycle assay) by using a Cell Cycle Detection Kit (Keygentec, Nanjing, China) according to the instruction of the manufacturer. Flow cytometry analysis for cell cycle was performed using the EPICS Elite ESP high-performance cell sorter (Coulter Electronics Ltd., England, UK), and a minimum of 20,000 events were collected for each sample. The raw collected data were analyzed by ModFit LT (version 2.0; Verity Software) to determine cell cycle distribution.

### 2.5. Immunofluorescent Chemistry and Confocal Microscopy Assays

Cells cultured in 12-well plates with coverslips were treated with phloretin (0, 20, 50, and 100 *μ*M). After 24 h, cells grown on coverslips were washed by 1× PBS and fixed with 2% paraformaldehyde for 15 min at room temperature, and then permeabilized by incubating with 0.2% Triton X-100 for 15 min. Next, cells were washed three times in 1× PBS and incubated in blocking buffer (3% bovine serum albumin in 1× PBS) for 1 h at room temperature. After blocking, cells were incubated with Sp1 and Sp3/4 antibody overnight at 4°C. Cells were washed with 1× PBS for three times (5 min each) and then incubated with anti-rabbit IgG-FITC secondary antibody (Santa Cruz, CA) in the dark at room temperature for 1 h. Finally, coverslips were washed with 1× PBS and stained with DAPI in the dark for 8 min. Fluorescent images were captured using a confocal microscope (Nikon, Sendai, Japan).

### 2.6. Plasmid Transfection and Dual-Luciferase Reporter Assay

Cells were plated in 12-well plates overnight, and the plasmids were transfected by using Lipofectamine 3000 (Invitrogen, Carlsbad, CA, USA). The nucleolin expression plasmid and luciferase reporter plasmids of Sp1-targeted genes were normalized to 1 *μ*g per well. At 4 h after transfection, the transfection mix of each well was replaced with complete media containing different concentrations of phloretin (0, 20, 50, and 100 *μ*M) for 24 h. Cells were then harvested and lysed for dual-luciferase assay according to the kit instruction (Beyotime Biotechnology). Relative luciferase units and fold inductions (compared with relative luciferase of control) were calculated by using the measured luciferase values.

### 2.7. Reverse Transcription-Polymerase Chain Reaction (RT-PCR)

Cells were cultured and treated with different concentrations of phloretin (0, 20, 50, and 100 *μ*M). After 24 h, cells were harvested for the extraction of total RNA and RT-PCR (*β*-actin as an internal control) according to the manufacturer's instructions. The primers used for PCR are listed in Table 1.

### 2.8. Experiments of Cytosolic and Nuclear Extracts

**Figure 1:**
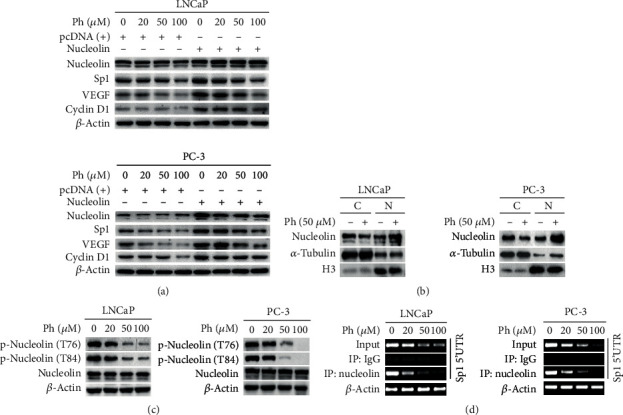
Phloretin decreased Sp1 level by inhibiting nucleolin binding to 5′-UTR of Sp1 mRNA via decreasing the levels of p-Nucleolin(T76 and T84) and regulating the nuclear and cytoplasmic distribution of nucleolin. (a) Cells were treated with different concentrations of phloretin and/or cotransfected with nucleolin expression plasmid, and then cells were harvested for western blotting assay to detect the levels of nucleolin, Sp1, VEGF, and Cyclin D1. (b) LNCaP and PC-3 cells were treated with/without phloretin (50 *μ*M) for 24 h and then harvested for nucleus and cytoplasm separation experiment and western blot assays to check the distribution of nucleolin in the cytoplasm (C) and the nucleus (N). (c) LNCaP and PC-3 cells were treated with phloretin (0, 20, 50, and 100 *μ*M) for 24 h and then harvested for western blot assay to check the levels of nucleolin, p-Nucleolin(T76), p-Nucleolin(T84), and *β*-actin (loading control). (d) LNCaP and PC-3 cells were treated with phloretin (0, 20, 50, and 100 *μ*M) for 24 h and then collected for RT-PCR (*β*-actin as internal control and Sp1 5′-UTR as input) and RNA-IP with IgG and anti-nucleolin antibody to check the binding levels of nucleolin to 5′-UTR of Sp1 mRNA. The RT-PCR products of mRNAs (including internal control *β*-actin mRNA, input Sp1 mRNA, and nucleolin-bound Sp1 mRNAs in the products of RNA-IP with IgG and anti-nucleolin antibody) were assayed by agarose gel electrophoresis.

Cells cultured in 60 mm plates were treated with phloretin as indicated in Figure 1(a). After 24 h, cells were washed with 1× PBS and harvested for extractions of cytosolic and nuclear proteins by using a Nuclear and Cytoplasmic Protein Extraction Kit (Beyotime Biotechnology). The cytosolic and nuclear extracts were then analyzed by western blot.

### 2.9. RNA Immunoprecipitation Assay (RIP Assay) and Western Blotting Analysis

Cells were treated with phloretin as indicated in Figure 1(c) and then harvested and lysed with lysis buffer (10 mM HEPES, pH 8.0, 40 mM KCl, 3 mM MgCl_2_, 5% glycerol, 0.5% NP-40, and 1 U/*μ*l RNaseOUT) for 30 min on ice. The cell lysates were divided into two parts: one part was for extracting the total RNA and then doing RT-PCR for Sp1 5′-UTR (5′-UTR of Sp1 mRNA, as input) and *β*-actin (as internal control); another part was for RNA immunoprecipitation (RIP) by incubating with IgG (Beyotime Biotechnology) and anti-nucleolin antibody, and then protein A/G agarose beads (Santa Cruz Biotechnology) at 4°C overnight. Immunoprecipitated complexes were washed with lysis buffer and RNA was extracted for RT-PCR of Sp1 5′-UTR. The RT-PCR products were detected with agarose gel electrophoresis. PCR primers specific for Sp1 5′-UTR were forward, 5′-ACT AGT AGC GAG TCT TGC CAT TGG -3′; reverse, 5′-GG CGC CGG TGG CAG CTG AGG GAC A-3′.

For western blotting analysis, cells were harvested and lysed in radio immunoprecipitation assay (RIPA) buffer (Beyotime Biotechnology) by adding Roche complete protease inhibitor cocktail. After centrifugation and protein quantification, the supernatants (containing total protein 15-30 *μ*g) were submitted to western blotting assay with related primary antibodies.

### 2.10. Antitumor Assay of Phloretin *In Vivo*

PC-3 cells were calculated by using trypan blue, and finally 2 × 10^6^ cells in 100 *μ*l 1× PBS were subcutaneously injected into male nude mice (4-5 weeks, supplied by the animal center in the College of Medicine, Nanjing University, Nanjing, China). After tumors grew to 24-30 mm^3^, mice were randomly divided into four groups (5 mice in each group) and treated every two days by intragastrical administration with 1× PBS (NC group), 5-FU (PC group, 20 mg/kg), and phloretin (including the LD group, low dose of 10 mg/kg; the HD group, high-dose of 50 mg/kg) for six weeks. After 42 days, mice were sacrificed and the subcutaneous tumors were isolated and weighted, and then the tumors were equally dissected into two parts. One part of the tumor tissues was stored at -80°C for western blot assay and another part was formalin fixed and paraffin embedded for immunohistochemistry.

### 2.11. Immunohistochemistry Analysis

The formalin-fixed tissues were sectioned in 4-5 *μ*m thick. Each tissue section was deparaffinized and rehydrated with upgraded ethanol, and then tissue sections were boiled in EDTA for 15 min, quenched with 0.3% hydrogen peroxide solution for 10 min at room temperature, and blocked with BSA in PBS for 30 min. The sections were subsequently incubated with special primary antibodies as indicated in figures overnight at 4°C and then counterstained with hematoxylin. Antibody binding was detected with an Envision Detection Kit, Peroxidase/DAB, Rabbit/Mouse (Gene Tech, Shanghai, China). The expression levels of specific proteins were observed and photographed under a microscope at a magnification of 400x (CTR 6000; Leica, Wetzlar, Germany).

### 2.12. Statistical Analysis

All data were expressed as the means ± SD and analyzed using Student's *t*-test. Comparison between groups was made by the Dunnett test of SPSS in figures. A *P* value of <0.05 was statistically significant. All experiments were replicated three times.

## 3. Results

### 3.1. Phloretin Induced Morphological Changes and Inhibited Cell Viability in Prostate Cancer Cells

**Figure 2:**
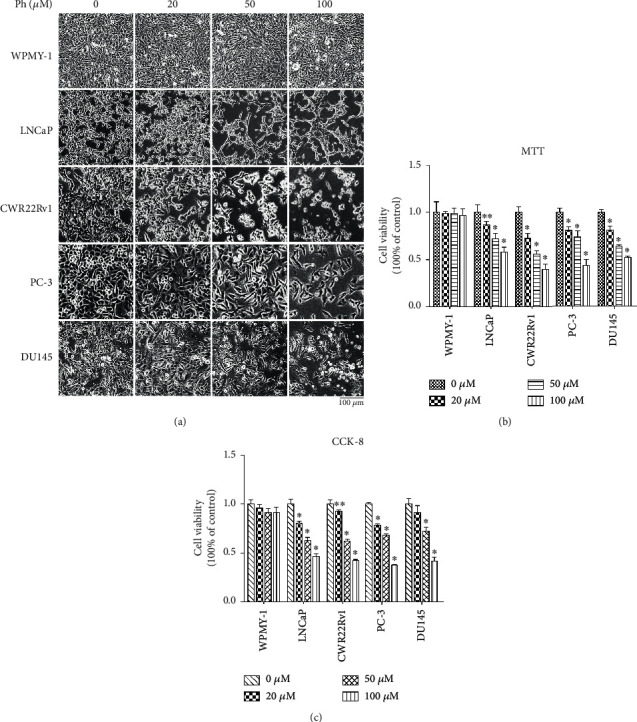
Phloretin treatment induced morphological changes and decreased cell viability in prostate cancer cells. (a) After treating with different concentrations of phloretin (0, 20, 50, and 100 *μ*M) in WPMY-1, LNCaP, CWR22Rv1, PC-3, and DU145 cells (2 × 10^5^ cells/well) for 24 h, cell morphological changes were observed and photographed with microscope (Nikon microscope, Japan) under 10x magnification. (b, c) WPMY-1, LNCaP, CWR22Rv1, PC-3, and DU145 cells were treated with different concentrations of phloretin (0, 20, 50, and 100 *μ*M) for 24 h, and then cells were harvested for MTT and CCK-8 assays, respectively. The percentages of cell viabilities were calculated by comparing with control samples. ^∗^*P* < 0.01, ^∗∗^*P* < 0.05.

To examine the effect of phloretin on cell viability, PCa cells (including LNCaP, CWR22Rv1, PC-3, and DU145 cells) and normal prostate epithelial cells (WPMY-1) were cultured and treated with different concentrations of phloretin (0, 20, 50, and 100 *μ*M) for 24 h, and then cell morphology and cell viability were photographed by microscopy and determined by MTT and CCK-8 assays. As shown in Figure 2(a), phloretin significantly induced PCa cell morphological changes (such as obviously shrunken, rounded, and even floated) in a dose-dependent manner, whereas no distinctly changes in normal WPMY-1 cells even treated with 100 *μ*M of phloretin. MTT and CCK-8 assays showed that phloretin inhibited the viability of PCa cells in a dose-dependent manner, while there is almost no effect on WPMY-1 cells (Figures 2(b) and 2(c)). All the results indicated that phloretin could inhibit cell growth, while there is no obvious effect on normal prostate cells.

### 3.2. Phloretin Induced Cell Cycle Arrest and Apoptosis in PCa Cells

**Figure 3:**
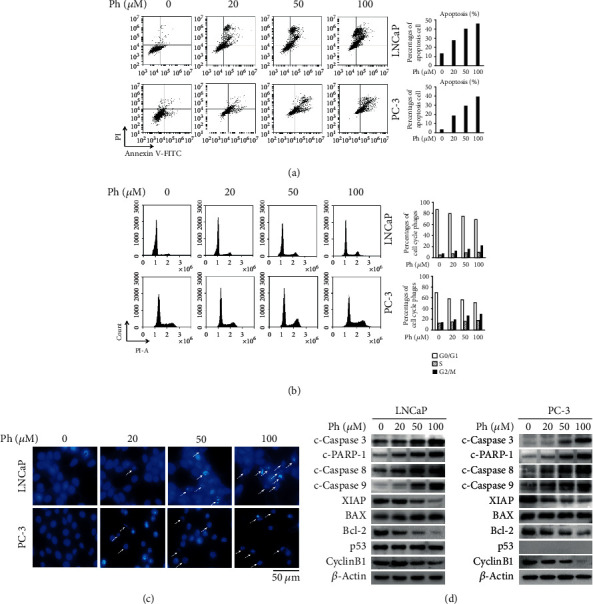
Phloretin induced cell apoptosis and cell cycle arrest in PCa cells. (a, b) LNCaP and PC-3 cells were treated with phloretin (0, 20, 50, and 100 *μ*M) for 24 h and then harvested for flow cytometry analysis to check cell cycle and apoptosis. Percentages of cell cycle phages and apoptosis were quantified, respectively. (c) LNCaP and PC-3 cells were treated with phloretin (0, 20, 50, and 100 *μ*M) for 24 h, and then cells were stained by DAPI, observed, and photographed with fluorescence microscope under 40x magnification. (d) LNCaP and PC-3 cells were treated with phloretin (0, 20, 50, and 100 *μ*M) and then harvested for western blot assay to check the protein levels of c-Caspase 3, c-PARP-1, c-Caspase 8, c-Caspase 9, Bcl-2, BAX, XIAP, p53, Cyclin B1, and *β*-actin (loading control).

LNCaP and PC-3 cells were cultured and treated with the different concentrations of phloretin (0, 20, 50, and 100 *μ*M). After 24 h, cells were treated for flow cytometry analysis and DAPI staining assay. As shown in Figure 3(a), phloretin treatment induced cell apoptosis in a concentration-dependent manner in both LNCaP and PC-3 cells. DAPI staining assay showed that phloretin treatment induced cell shrivelled to rupture and nuclearly broken into pieces in a concentration-dependent manner (pointed by the white arrow) and resulted in cell apoptosis in LNCaP and PC-3 cells (Figure 3(c)). In addition, cell cycle analysis demonstrated that phloretin treatment induced cell cycle arrest in G2/M phase in a concentration-dependent manner in both cell lines (Figure 3(b)).

Western blot data further identified that phloretin treatment in LNCaP and PC-3 cells decreased the protein levels of Cyclin B1, XIAP, and Bcl-2, while increased the protein levels of c-Caspase 3 (cleaved Caspase 3), c-PARP-1 (cleaved PARP-1), c-Caspase 8 (cleaved Caspase 8), and c-Caspase 9 (cleaved Caspase 9). The change degrees were all in a phloretin dose-dependent manner (Figure 3(d)). Although phloretin increased the level of BAX in a dose-dependent manner only in LNCaP cells (no changes in PC-3 cells), the BAX/Bcl-2 ratios in both cell lines were upregulated by phloretin in a dose-dependent manner. Furthermore, we found that phloretin treatment did not change the protein level of p53 in LNCaP cells (undetectable in PC-3 cells) (Figure 3(d)), suggesting that phloretin-induced PCa cell apoptosis was p53 independent.

### 3.3. Phloretin Inhibited the Activation of EGFR and Its Downstream PI3K/AKT and MEK/ERK Pathways and Then Decreased the Activities of GSK-3*β* and Sp1

**Figure 4:**
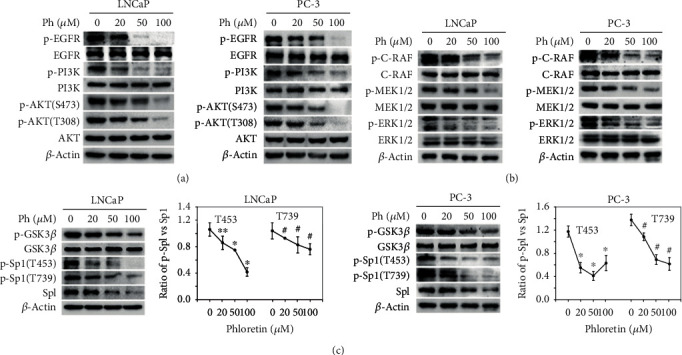
Phloretin inhibited the activities of EGFR and its downstream PI3K/AKT, MEK/ERK, and GSK3*β* pathways. (a) LNCaP and PC-3 cells were incubated with phloretin (0, 20, 50, and 100 *μ*M) for 24 h and then harvested for western blot assays to check the protein levels of p-EGFR(Y1173), EGFR, p-PI3K, PI3K, p-AKT(S473), p-AKT(T308), AKT, p-GSK3*β*(S9), GSK3*β*, and *β*-actin (loading control). (b) LNCaP and PC-3 cells were treated with phloretin as above and then harvested for western blot assays to check the protein levels of p-C-RAF(S338), C-RAF, p-MEK(S217/S221), MEK, p-ERK1/2(T202/Y204), ERK1/2, and *β*-actin (loading control). (c) LNCaP and PC-3 cells were treated with phloretin and then harvested for western blot assay to check the protein levels of p-Sp1(T453), p-Sp1(T739), Sp1, and *β*-actin (loading control); the ratio of p-Sp1 levels vs. total Sp1 levels was quantified using ImageJ software. ^∗^*P* < 0.01; ^∗∗^*P* < 0.05; ^#^*P* < 0.01.

In exploring the molecular mechanism of phloretin-induced cell growth inhibition, cell cycle arrest, and apoptosis in PCa cells, we found that phloretin treatment substantially downregulated the autophosphorylation levels of EGFR at Y1173, but not the total protein level of EGFR (Figure 4(a)), suggesting the activity of EGFR was inhibited by phloretin (it is the same as isorhapontigenin treatment in PCa cells we reported previously [35]). As the downstream of EGFR, the PI3K/AKT and MEK/ERK1/2 pathways have been reported to play the crucial roles in regulating cell survival, growth, migration, and invasion [20, 21]. From the western blot data, we also found that phloretin downregulated the levels of p-PI3K, p-AKT(S473), p-AKT(T308), p-C-RAF, p-MEK, and p-ERK1/2 in a concentration-dependent manner, while there is almost no effect on the total protein levels of PI3K, AKT, RAF, MEK1/2, and ERK1/2 in PCa cells (Figures 4(a) and 4(b)).

GSK3*β* is a downstream target of AKT and inactivated by AKT-induced phosphorylation of GSK3*β* proteins at Ser9 [25]. Our results showed that phloretin treatment in PCa cells decreased the phosphorylation level of GSK3*β* at Ser9 in a concentration-dependent manner, while the total protein level of GSK3*β* unchanged (Figure 4(c)), suggesting that phloretin increased the activity of GSK3*β* via inhibiting the PI3K/AKT pathway.

As reported, the activity of Sp1 was regulated by AKT, ERK1/2, and GSK3*β*, respectively. AKT could upregulate the expression of *Sp1* gene [36, 37], ERK1/2 could phosphorylate Sp1 at Thr453/Thr739 and then facilitate its binding to promoters of the targeted genes [26, 27], and GSK3*β* could induce the degradation of Sp1 proteins [24, 25]. Here, phloretin treatment in LNCaP and PC-3 cells decreased the total protein level of Sp1 and the phosphorylation levels of Sp1 at Thr453/Thr739 in a concentration-dependent manner, and the quantification data of the ratios of p-Sp1/Sp1 (including p-Sp1 at both T453 and T739) further demonstrated that phloretin-induced decrease of p-Sp1(T453/T739) levels was also concentration dependent (Figure 4(c)). Therefore, phloretin-induced inhibition of AKT and ERK1/2 inevitably inhibited *Sp1* expression, decreased the phosphorylation of Sp1(T453/T739) and GSK3*β*(S9), induced Sp1 degradation, and finally decreased Sp1 activity and impaired Sp1 binding to the promoters of its downstream genes.

Together, phloretin inhibited AKT and ERK1/2 and activated GSK3*β*, and subsequently decreased the transcriptional activity of Sp1 by downregulating the expression of *Sp1* gene, inducing the degradation of Sp1 and decreasing Sp1-binding to the promoters of its target genes in PCa cells.

### 3.4. Phloretin Decreased the Expression of Sp1-Targeted Genes in PCa Cells

**Figure 5:**
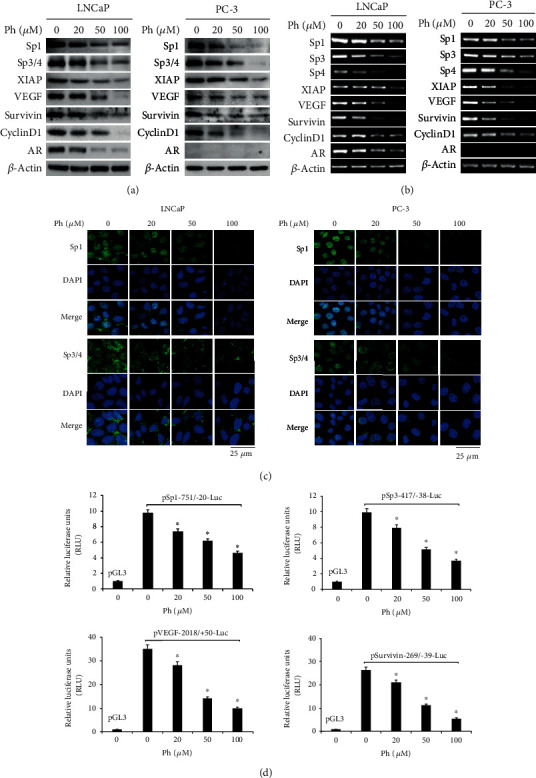
Phloretin decreased the expression of Sp1-targeted genes by downregulating Sp1 levels in PCa cells. (a, b) LNCaP and PC-3 cells were treated with phloretin (0, 20, 50, and 100 *μ*M) for 24 h and then harvested for western blot (a) and RT-PCR (b) assays to check the expressions of *Sp1* and its target genes (including *Sp3/4*, *XIAP*, *VEGF*, *Survivin*, *Cyclin D1*, and *AR*) in both mRNA and protein levels (*β*-actin as control). (c) LNCaP and PC-3 cells were cultured in 12-well plates with cover slips and then treated with phloretin (0, 20, 50, and 100 *μ*M) for 24 h. Cells grown on cover slips were treated for immunofluorescent confocal analysis with Sp1 and Sp3/4 antibodies (the cell nucleus was stained with DAPI). (d) PC-3 cells were transfected with pSp1-751/-20-Luc, pSp3-417/-38-Luc, pVEGF-2018/+50-Luc, pSurvivin-269/-39-Luc, and pGL3-basic and treated with phloretin (0, 20, 50, and 100 *μ*M), and then luciferase activities were measured by using the Dual-Luciferase Reporter Assay Kit. The relative luciferase units (RLU) were calculated. ^∗^*P* < 0.01.

As a transcription factor, Sp1 could regulate the expression of multiple genes, including *Sp3/4*, *XIAP*, *VEGF*, *Survivin*, *Cyclin D1*, *AR*, and *Sp1* itself [16–19, 32, 38–41]. To identify the effect of phloretin on these Sp1-targeted genes, LNCaP and PC-3 cells were cultured and treated with different concentrations of phloretin (0, 20, 50, and 100 *μ*M). After 24 h, cells were harvested for both western blot assay and RT-PCR assay as indicated in Figures 5(a) and 5(b). The results showed that phloretin treatment in LNCaP and PC-3 cells decreased the expression of *Sp1* and its target genes (including *Sp3/4*, *XIAP*, *VEGF*, *Survivin*, *Cyclin D1*, and *AR*) in both protein and mRNA levels in a concentration-dependent manner (Figures 5(a) and 5(b)). Further, the confocal data showed that phloretin treatment in LNCaP and PC-3 cells obviously decreased the protein levels of Sp1 and Sp3/4 in a phloretin concentration-dependent manner (Figure 5(c)).

Additionally, data of dual luciferase reporter assay also showed that phloretin treatment decreased the luciferase activities in PC-3 cells transfected with plasmids pSp1(-751/-20)-Luc, pSp3(-417/-38)-Luc, pVEGF(-2018/+50)-Luc, and pSurvivin(-269/-39)-Luc in a concentration-dependent manner (Figure 5(d)).

These results demonstrated that phloretin treatment in PCa cells downregulated the expression of Sp1-targeted genes by decreasing the transcription activity of Sp1.

### 3.5. Phloretin Decreased the Level of Sp1 by Increasing the Degradation of Sp1 Protein in PCa Cells

**Figure 6:**
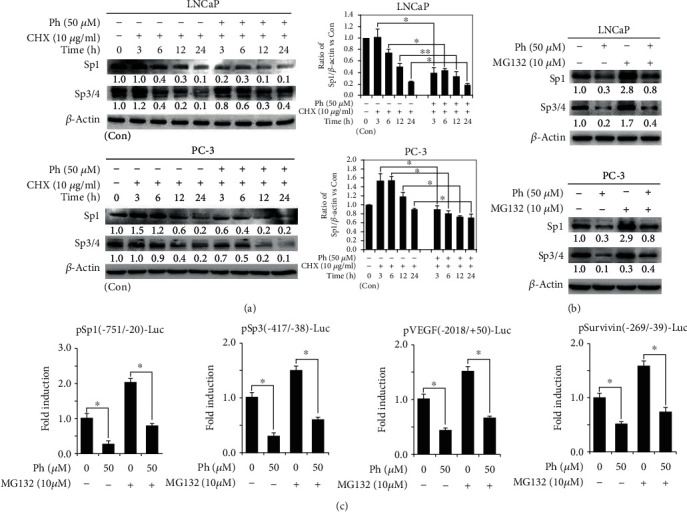
Phloretin induced the degradation of Sp1 protein in PCa cells. (a) Cells were treated with 10 *μ*g/ml CHX alone or cotreated with 10 *μ*g/ml CHX and 50 *μ*M phloretin (CHX pretreated for 30 min) for western blot assays to detect the protein levels of Sp1 and Sp3/4. The quantification: first, the densities of bands of Sp1 and *β*-actin were quantified using ImageJ software, and the ratios of Sp1/*β*-actin (the Sp1 and *β*-actin with no treatment of phloretin and CHX as control) were calculated; second, the ratios of Sp1/*β*-actin vs. Con were obtained by using the values of Sp1/*β*-actin vs. the value of control Sp1/*β*-actin (control Sp1/*β*-actin vs. control Sp1/*β*-actin as 1). (b) Cells were treated with phloretin (50 *μ*M) and/or MG132 (10 *μ*M), and then cells were harvested for western blotting assay to detect the protein levels of Sp1 and Sp3/4. All western blotting assays used *β*-actin as loading control. (c) PC-3 cells were transfected with the various luciferase constructs and treated with phloretin (50 *μ*M) or MG132 (10 *μ*M) alone, or cotreated with phloretin (50 *μ*M) and MG132 (10 *μ*M), and then dual-luciferase activities (Firefly and Renilla) were measured. The fold inductions of luciferases were calculated by using relative luciferases (the relative luciferase of control as 1). ^∗^*P* < 0.01; ^∗∗^*P* < 0.05. Con: control.

To identify the degradation of Sp1 protein by phloretin-induced inhibition of PI3K/AKT and activation of GSK3*β*, LNCaP and PC-3 cells were cultured and treated with CHX (10 *μ*g/ml) and/or phloretin (50 *μ*M) for different time as indicated Figure 6(a), and then cells were harvested for western blot assay. The data showed that treatment with CHX alone caused the decrease of Sp1 protein level in a time-dependent manner; cotreatment with CHX and phloretin resulted in a more decrease of Sp1 protein level. The quantification data also demonstrated that the levels of Sp1 in CHX-treated cells were further decreased by phloretin treatment in a time-dependent manner, suggesting that phloretin could induce the degradation of Sp1 proteins. In addition, the Sp1-targeted genes Sp3/4 were also decreased with the downregulation of Sp1 (Figure 6(a)).

To further identify phloretin-induced degradation of Sp1, LNCaP and PC-3 cells were cultured and treated with phloretin (50 *μ*M) and/or MG132 (10 *μ*M) as indicated in Figure 6(b) and then harvested for western blot assay. Our data showed that phloretin-induced decrease of Sp1 and Sp3/4 could be partially rescued by cotreated MG132 (Figure 6(b)). Moreover, dual-luciferase assay showed that MG132 treatment could also partially reverse the phloretin-induced decrease of the luciferase activities in PC-3 cells transfected with pSp1(-751/-20)-Luc, Sp3(-417/-38)-Luc, VEGF(-2018/+50)-Luc, and Survivin(-269/-39)-Luc, respectively (Figures 6(b) and 6(c)). It demonstrated that phloretin induced the protein level decrease of Sp1 and its target genes were not only decreasing Sp1 gene expression and Sp1 mRNA translation but also inducing Sp1 protein degradation.

### 3.6. Phloretin Decreased the Sp1 Levels by Decreasing Cytoplasmic Distribution of Nucleolin and Then Decreasing the Binding of Nucleolin to 5′-UTR of Sp1 mRNA

To identify the effect of nucleolin on phloretin-induced decrease of Sp1 proteins in PCa cells, LNCaP and PC-3 cells were transfected with expression plasmid of nucleolin and treated with phloretin as indicated in Figure 1(a) and then checked the protein levels of Sp1 and its downstream proteins, including VEGF and Cyclin D1. From the experimental data, we found that overexpression of nucleolin upregulated the protein levels of Sp1 and its downstream proteins in both LNCaP and PC-3 cells, and phloretin treatment in PCa cells obviously suppressed nucleolin-induced upregulation of protein levels of Sp1 and its targeted proteins (Figure 1(a)).

It is reported that nucleolin could be recruited to the 5′-UTR of Sp1 mRNA in the cytoplasm as an IRES (internal ribosomal entry site, nucleolin binding site is +1/250 bp of 5′-UTR of Sp1 mRNA) transacting factor to enhance the translation of Sp1 mRNA during lung cancer formation [31], and PI3K/AKT-induced phosphorylation of nucleolin at Thr76 and Thr84 promoted nucleolin translocation from the nucleus to the cytoplasm in the cells of colorectal carcinoma [30]. To identify the effect of phloretin on the phosphorylation of nucleolin at Thr76 and Thr84 and the distribution of nucleolin in the nucleus and the cytoplasm, LNCaP and PC-3 cells were incubated with 50 *μ*M or different concentrations of phloretin (as indicated in Figures 1(b) and 1(c)) for 24 h. Then, cells were harvested for nucleus/cytoplasm separation and/or western blot assays to detect the nucleus/cytoplasm distribution and phosphorylation (Thr76/Thr84) levels of nucleolin. From the results, we found that the protein levels of nucleolin were decreased in cytoplasm and increased in the nucleus with the treatment of phloretin (Figure 1(b)). Levels of p-Nucleolin(Thr76) and p-Nucleolin(Thr84) were distinctly decreased by the treated phloretin in a concentration-dependent manner, while the total protein levels of nucleolin were almost not changed in LNCaP and PC-3 cells (Figure 1(c)).

In addition, RNA-IP experiment was employed to identify the effect of phloretin on nucleolin binding to 5′-UTR of Sp1 mRNA. LNCaP and PC-3 cells (treated with different concentrations of phloretin as indicated in Figure 1(d)) were harvested for direct RT-PCR of 5′-UTR of Sp1 mRNA and for RNA-IP with IgG/nucleolin antibodies. Then, the RNAs of IP were also extracted for RT-PCR of 5′-UTR of Sp1 mRNA. The direct RT-PCR data showed that the mRNA levels of *β*-actin (as internal control) were not changed with the treatment of phloretin, while the total levels of 5′-UTR of Sp1 mRNA (as input) were decreased with the treatment of phloretin in a concentration-dependent manner in both PCa cell lines (Figure 1(d)). The RNA-IP experimental data showed that the levels of nucleolin-bound 5′-UTR of Sp1 mRNA were obviously decreased with the treatment of phloretin in a concentration-dependent manner in both PCa cell lines, and the decreasing degree was a little higher than that of input of 5′-UTR (Figure 1(d)). Besides, no RT-PCR products were detected in the negative control samples of RNA-IP with IgG (Figure 1(d)).

These results suggested that phloretin treatment in PCa cells decreased the levels of cytoplasmic nucleolin via downregulating the phosphorylation levels of nucleolin at Thr76 and Thr84 and then resulted in the reduction of nucleolin binding to 5′-UTR of Sp1 mRNA in the cytoplasm. It might inevitably lead to the decrease of nucleolin-induced Sp1 mRNA translation.

### 3.7. Phloretin Suppressed Tumor Growth and Induced Apoptosis of Prostate Cancer Cells *In Vivo*

**Figure 7:**
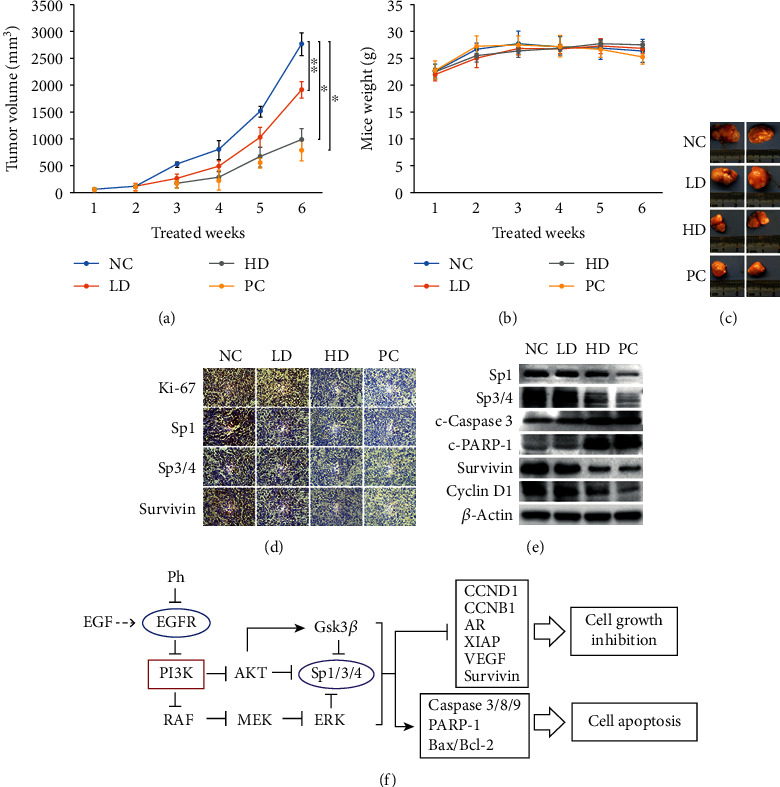
Phloretin suppressed PC-3 cell xenograft tumor growth and induced PC-3 cell apoptosis in nude mice. The nude mice with PC-3 cell xenograft tumors in the subcutaneous tissue (~30-50 mm^3^) were treated intraperitoneally with phloretin (low-dose 10 mg/kg and high-dose 50 mg/kg), 1× PBS (as negative control), and 5-FU (as positive control) every two days for 6 weeks. (a) Tumor volumes of the PBS group (negative control), the low-dose phloretin group (low Ph), the high-dose phloretin group (high Ph), and the 5-FU group (positive control) were measured every week, and the average tumor volumes of each group were calculated. ^∗^*P* < 0.01, ^∗∗^*P* < 0.05. (b) Mouse weights were measured every week and the average mouse weights of four groups were calculated. (c) At the end of the experiment, mice were sacrificed, and subcutaneous tumors were isolated and photographed. (d) The protein levels of Ki-67, Sp1, Sp3/4, and Survivin in tumor tissues were checked by using IHC assay. (e) The protein levels of Sp1, Sp3/4, c-Caspase 3, c-PARP-1, Survivin, and Cyclin D1 in tumor tissues were determined by using western blot assay. (f) Schematic diagram of signal pathways in phloretin-induced cell growth inhibition and apoptosis in human prostate cancer cells. NC: negative control; LD: low dose; HD: high dose; PC: positive control.

To investigate the efficacy of phloretin on prostate cancer cells *in vivo*, subcutaneous xenotransplanted tumor models of PC-3 cell in nude mice were established to evaluate the inhibition of tumor growth by phloretin treatment. Compared with the negative control group, our results showed that phloretin treatment *in vivo* inhibited growth of the transplanted prostate tumors in a time-dependent manner in both the high-dose and low-dose phloretin-treated groups, while the body weights of mice had no significant changes in all groups (Figures 7(a) and 7(b)). At the end of experiments, the tumor volumes of mice in the high-dose (HD) group were much smaller than those in both the negative control (NC) and low-dose (LD) groups and almost the same as those in the positive control (PC) group (Figures 7(a) and 7(c)).

Immunohistochemistry (IHC) data showed that the levels of Ki-67 (marker of cell proliferation), Sp1, Sp3/4, and Survivin in the cells of PC-3-transplanted tumor tissues were all decreased with the treatment of phloretin, and these protein levels in the tumor cells of the HD group were almost the same as those of the PC group (5-FU treatment) (Figure 7(d)). In addition, the western blot data of PC-3-transplanted tumor tissues demonstrated that the protein levels of Sp1, Sp3/4, Survivin, and Cyclin D1 were decreased, while the protein levels of c-Caspase 3 and c-PARP-1 were increased in the HD and PC groups (compared with the NC and LD groups) (Figure 7(e)).

These results demonstrated that phloretin treatment could also inhibit PC-3 cell growth and induce PC-3 cell apoptosis *in vivo*, and the high-dose phloretin had a greater inhibition efficacy on the growth of PCa cell-transplanted tumors by comparing with low-dose phloretin *in vivo*.

## 4. Discussion

Phloretin has been recently attracted extensive interest and extensively studied in treating many diseases including in anticancer research. Phloretin could induce apoptosis of many types of cancer cells *in vitro* and inhibit growth of the transplanted tumors of multiple human cancer cell lines *in vivo*[5, 7, 42]. However, the molecular mechanism of the anticancer effect of phloretin in prostate cancer remained unclear. In this study, we reported that phloretin could inhibit the activation of EGFR and its downstream signal pathways, including the PI3K/AKT, MEK/ERK1/2, and GSK3*β* pathways. Inhibition of these pathways further inhibited the activation of Sp1 and subsequently resulted in the downregulation of Sp3/4 proteins, cell growth, and antiapoptosis-related proteins (including Cyclin D1, Cyclin B1, Bcl-2, Survivin, AR, VEGF, and XIAP) and the upregulation of cell apoptosis-related proteins (including c-Caspase 3, c-Caspase 8, c-Caspase 9, and c-PARP-1), and finally resulted in cell growth inhibition and apoptosis in prostate cancer cells both *in vitro* and *in vivo* (Figure 7(f)).

Usually, p53 is the key protein in cell growth inhibition and cell apoptosis promotion involved in the inhibition of pathways that related to cell cycle and the activation of pathways that related to cell apoptosis [43, 44]. In current study, the protein levels of p53 were almost not changed in LNCaP cells and almost not detected in PC-3 cells (Figure 3(d)). In addition, the phosphorylation levels of p53(Ser15) in LNCaP cells which related to the activity of p53 protein were decreased with the treatment of phloretin (data not shown). It indicated that p53 was not the key factor in phloretin-induced cell growth inhibition and apoptosis in prostate cancer cells. Thus, our experimental results indicated that phloretin-induced cell apoptosis was in a p53-independent manner in PCa cells. Based on the capability of Sp1 in modulating the expression of Cyclin B1 gene [45], we could conclude that phloretin-induced downregulation of cyclin B1 and cell cycle arrest at G2/M phase in PCa cells was not via the p53/cyclin B1 pathway, but via the Sp1/cyclin B1 pathway.

Nucleolin, the RNA-binding protein, is involved in mRNA processing including the regulation of mRNA stability and translational efficiency [46]. During lung cancer formation, nucleolin was recruited to the 5′-UTR of Sp1 mRNA to enhance cap-independent translational activity in the cytoplasm. Phosphorylation of nucleolin was also important to increase the distribution of nucleolin protein in the cytoplasm and enhance nucleolin binding to 5′-UTR of Sp1 mRNA [30, 31]. Our results here identified that phloretin treatment in PCa cells downregulated the phosphorylation levels of nucleolin and resulted in the decreased distribution of nucleolin protein in the cytoplasm and reduced nucleolin binding to 5′-UTR of Sp1 mRNA, and finally decreased the translational efficiency of Sp1 mRNA. Of course, more details of the molecular mechanism of the relationship between phloretin and nucleolin needed to be further explored.

Sp1, usually overexpressed in many human tumors and cancer cell lines [8, 9], plays an important role in tumorigenesis and cancer progress and is a potential target for development of drugs in cancer chemotherapy [47]. Regulation of Sp1 activity should be an effective strategy for cancer drug screening. As reported, Sp1 is positively regulated by AKT and ERK and negatively regulated by GSK3*β*, and GSK3*β* is negatively regulated by the PI3K/AKT pathway again [24–27, 36, 37]. Our study demonstrated that phloretin-induced inhibition of Sp1 in PCa cells included at least three pathways: first, phloretin induced inhibition of EGFR/PI3K/AKT and then downregulated expression of *Sp1* gene (phloretin-EGFR/PI3K/AKT-Sp1 pathway); second, phloretin induced inhibition of EGFR/PI3K/AKT and then decreased the phosphorylation levels of GSK3*β*(S9) and activated GSK3*β*, and finally induced the degradation of Sp1 proteins (by combined with reported results in [48]); and third, phloretin induced inhibition of EGFR/PI3K/AKT and then downregulated the phosphorylation levels of nucleolin(Thr76/Thr84) and might suppress the translation of Sp1 mRNA (by combined with reported results in [31]). Of course, the detailed molecular mechanisms of phloretin in regulating Sps needed to be further studied in the future studies.

In conclusion, phloretin treatment in PCa cells downregulated the protein levels of Sp1 by inhibiting the expression of Sp1 genes, increasing the degradation of Sp1 proteins and suppressing the translation of Sp1 mRNAs via inhibiting the activity of EGFR and its downstream pathways, including the PI3K/AKT, MEK/ERK, AKT/GSK3*β*, and AKT/nucleolin pathways, and finally induced cell cycle arrest, cell growth inhibition, and apoptosis in PCa cells by decreasing the levels of cell cycle/growth-related proteins and increasing the levels of cell apoptosis-related proteins. Our study suggested that phloretin had the potential to be a candidate compound to treat the patients with prostate cancer in clinic in the future.

## Figures and Tables

**Table 1:** The primers used for PCR.

Primers	Sequences (5′-3′)	Fragment size (bp)
*β*-Actin/forward	GAG CTA CGA GCT GCC TGA CG	416
*β*-Actin/reverse	CCT AGA AGC ATT TGC GGT GG	
Sp1/forward	CTG CTA TGC CAA ACC TAC TCC	415
Sp1/reverse	CCC TGT AGC CCA CTG ACC CT	
SP3/forward	CAA ACC TTA CTT GCC TCT GGA ACA C	329
SP3/reverse	TTA ATA TCA GGA GAA ACC CGC TCA C	
SP4/forward	AGA AGG AAG AGG CAG TAA TGA ACC A	108
SP4/reverse	CGA AGA TGT GCT CGT AAA TGA GAT G	
VEGF/forward	GAG GGC AGA ATC ATC ACG AA	436
VEGF/reverse	AGG CTC CAG GGC ATT AGA CA	
Survivin/forward	CAG ACT TGG CCC AGT GTT TCT	235
Survivin/reverse	TTC TCC GCA GTT TCC TCA A	
XIAP/forward	CCT TGT GAT CGT GCC TGG TC	315
XIAP/reverse	AGG GTC TTC ACT GGG CTT CC	
AR/forward	CGG ACG AGG ATG ACT CAG	457
AR/reverse	TCT TCA GTG CTC TTG CCT GC	
Cyclin D1/forward	CTG CGA AGT GGA AAC CAT CC	360
Cyclin D1/reverse	TAG ATG CAC AGC TTC TCG GC	

## Data Availability

The data used to support this study are available from the corresponding author upon request.
